# Bibliometric trends and patterns in Tasar silkworm (*Antheraea mylitta*) research: a data report (1980–2024)

**DOI:** 10.3389/finsc.2025.1533267

**Published:** 2025-04-30

**Authors:** J. Komal, R. Gowrisankar, Vishaka G. V., H. Nadaf, Ipsita Samal, Pasumarthi Venkata Dinesh Kumar, C. Selvaraj, B. Thirupam Reddy, T. Selvakumar, Deepak Kumar Mahanta, Tanmaya Kumar Bhoi

**Affiliations:** ^1^ Basic Seed Multiplication and Training Centre, Central Silk Board, Kharaswan, Jharkhand, India; ^2^ Basic Seed Multiplication and Training Centre, Central Silk Board, Nabarangpur, Odisha, India; ^3^ Basic Tasar Silkworm Seed Organization, Central Silk Board, Bilaspur, Chhattisgarh, India; ^4^ Indian Council of Agricultural Research – National Research Centre on Litchi, Muzaffarpur, Bihar, India; ^5^ Research Extension Centre, Central Silk Board, Hoshangabad, Madhya Pradesh, India; ^6^ Basic Seed Multiplication and Training Centre, Central Silk Board, Madhupur, Jharkhand, India; ^7^ Basic Seed Multiplication and Training Centre, Central Silk Board, Parsada, Chhattisgarh, India; ^8^ Forest Protection Division, Indian Council of Forestry Research and Education – Forest Research Institute (ICFRE-FRI), Dehradun, Uttarakhand, India; ^9^ Forest Protection Division, Indian Council of Forestry Research and Education – Arid Forest Research Institute (ICFRE-AFRI), Jodhpur, Rajasthan, India

**Keywords:** bibliometrics, Tasar silkworm, Scopus, Biblioshiny application, Bibliometrix Package, R, VOSviewer

## Abstract

This study presents a bibliometric analysis of publication trends in Tasar silkworm (*Antheraea mylitta*) research from 1980 to 2024. A comprehensive search was conducted using the Scopus database with keywords related to Tasar silkworm. A total of 741 relevant articles were identified and analyzed using VOSviewer, Bibliometrix, and Biblioshiny in R to examine statistical patterns. Over the decades, research focus has transitioned from fundamental silk characterization to biomedical applications, including tissue engineering, biodegradation studies, and antioxidant properties. Publication trends indicate peak research activity between 2007 and 2010, followed by a decline post-2018, likely due to shifting priorities toward commercially dominant silkworm species. Indian institutions have been the primary contributors, reflecting strong domestic expertise, while global collaborations remain limited. Keyword analysis highlights the growing interdisciplinary nature of Tasar silk research, extending into biomaterials and sustainable technology. Future research directions emphasize biotechnological advancements, biomedical applications, eco-friendly processing, climate resilience, and commercialization strategies. Strengthening international collaborations and integrating innovative technologies will be crucial for advancing Tasar silk research in both scientific and industrial domains.

## 1 Introduction

The Tasar silkworm (*Antheraea mylitta*) possesses considerable economic and ecological significance, especially in India, where it is essential for sustaining rural livelihoods and tribal communities (1). Tasar silk, characterized by its distinctive golden-brown color and coarse texture, is esteemed in both domestic and foreign markets. Tasar silkworms, in contrast to mulberry silk, are cultivated in their wild habitat, consuming a number of host plants primarily feeding on *Terminalia arjuna* (Arjun), *Terminalia tomentosa* (Asan) and *Shorea robusta* (Sal), thereby intertwining silk production with forest ecosystems (2). Tasar sericulture serves as both a source of income and a sustainable technique that fosters biodiversity protection and ecological equilibrium (3). The inherent wildness of the silkworm and its reliance on forest resources guarantee that Tasar silk manufacturing is environmentally sustainable, offering a renewable, biodegradable substitute for synthetic fibers, which is more important in the contemporary movement towards sustainable textiles (4).

Current developments in Tasar silkworm research indicate progress in critical domains, including genetic improvement, disease management, and sustainable host plant cultivation (5–9). Biotechnology has facilitated the development of silkworm lines with enhanced yield and disease resistance, while molecular research aims to elucidate the silkworm’s immune system and refine disease management strategies (10). There is increasing interest in improving agroforestry systems to improve host plant cultivation and investigating the potential of Tasar silk for industrial and biomedical uses (3, 11–13). Furthermore, since climate change impacts forest ecosystems, research on climate adaptation in Tasar sericulture is gaining significance (14). These advancements are establishing Tasar silk as an essential element in sustainable economic development and ecological conservation, in accordance with the increasing worldwide demand for eco-friendly products.

This study sought to conduct a bibliometric analysis of literature about the Tasar silkworm listed in the Scopus database. A quantitative methodology was employed to do a bibliometric study of published publications to attain this purpose. This bibliometric study seeks to deliver a thorough analysis of the research environment pertaining to Tasar silkworms, emphasizing the identification of publishing trends, research clusters, and emerging subjects. This study will analyze the volume and citation patterns of academic outputs to trace the historical development of research and identify present gaps and future trends in the area. This research enhances comprehension of the evolution of scientific investigation into Tasar silkworms and its significance in the larger framework of sustainable textiles, rural development, and conservation of biodiversity.

It is noteworthy that, as of now, no bibliometric analysis of Tasar silkworm research exists. Consequently, this study holds significant relevance at this juncture. This study aimed to address the following inquiries regarding tasar silkworm research:

1. What is the pace of increase for scientific production in tasar silkworm research every year?
2. What is the scientific output of authors? What are the most significant articles and sources, both internationally and locally, in tasar silkworm research?
3. Which themes are novel, emerging, developed, and underdeveloped in tasar silkworm research?
4. What is the country’s scientific production like? What is the extent of the collaborative network among authors, institutions, and nations in tasar silkworm research?
5. What are the top keywords, trends in topics, and co-occurrence network of keywords in tasar silkworm research?

## 2 Materials and methods

### 2.1 Retrieval of data

The study used a bibliometric approach for the identification, representation, visualization, and analysis of literary data. The bibliometric approach was developed by Allen Richard in 1969, building upon earlier contributions by Coles and Eales in 1917 (15). Bibliometric approaches are used to identify trends and developments across several scientific fields (16–19). Bibliometric analysis and meta-analysis use quantitative methods to reduce bias, while systematic literature reviews mostly rely on qualitative techniques, which may be prone to interpretative bias from scholars with diverse academic backgrounds (20). A range of databases is available for importing bibliographic data, such as Scopus, Web of Science (WoS), Dimensions, Cochrane Library, Lens, and PubMed, each possessing distinct features and functionalities. The Web of Science and Scopus are now the most extensively used literature databases across almost all fields (21). This study used a document search in the Scopus database (https://www.scopus.com), which generates a significant volume of papers and provides more citation-rich data (22). The present analysis omits items not indexed by Scopus. Scopus is a comprehensive database that enables scholars to assess and analyze papers, patents, clinical trials, and policy documents. We analyzed article titles, abstracts, and authors’ keywords. The search parameter was (TITLE-ABS-KEY (Tasar AND Silkworm) OR TITLE-ABS-KEY (Tasar AND Silk) OR TITLE-ABS-KEY (Tasar AND Moth) OR TITLE-ABS-KEY (Antheraea AND mylitta) OR TITLE-ABS-KEY (Tasar AND Vanya AND Silk)) AND PUBYEAR > 1979 AND PUBYEAR < 2025 AND (LIMIT-TO (DOCTYPE, “ar”) OR LIMIT-TO (DOCTYPE, “re”) OR LIMIT-TO (DOCTYPE, “cp”) OR LIMIT-TO (DOCTYPE, “ch”) OR LIMIT-TO (DOCTYPE, “no”) OR LIMIT-TO (DOCTYPE, “sh”) OR LIMIT-TO (DOCTYPE, “le”) OR LIMIT-TO (DOCTYPE, “cr”) OR LIMIT-TO (DOCTYPE, “bk”)) AND (LIMIT-TO (LANGUAGE, “English”))[ar: Articles; re: Review; cp: Conference paper; ch: Chapters; no: Notes; sh: Short surveys; le: Letter; cr: Conference review; bk: Book]. The search was narrowed by specifying a publication date range from 1980 to 2024. The data was downloaded on 24^th^ March, 2025, resulting in an initial selection of 766 articles.

### 2.2 Screening of data

The present investigation included original articles, review papers, books, book chapters, conference proceedings, and brief surveys. We performed a comprehensive assessment of all available data to identify research specifically focused on tasar silkworm. Only English-language articles are selected for the investigation. The redundant articles (Duplicate/irrelevant articles) have been removed from the dataset. Utilizing these inclusion and exclusion criteria, we identified 741 relevant scientific publications. The dataset included 741 documents, which served as the basis for the bibliometric analysis conducted in this study. **Supplementary Figure 1** illustrates the search algorithm used in this study to locate relevant publications from the Scopus database. The comprehensive bibliographic collection was acquired in.csv format from the Scopus database.

### 2.3 Analysis of data

The Bibliometrix R package (version R.4.4.1) was first installed and subsequently loaded in R Studio. The Biblioshiny program was started by inputting “Biblioshiny()” on the R command line. Biblioshiny is a web application that facilitates access to the Bibliometrix software in R. Bibliometrix offers many tools for comprehensive bibliometric analysis for scholars (23). Biblioshiny, a statistical software tool, was used for data mining in bibliometrics to ascertain the probability of simultaneous keyword occurrences in scientific works, therefore elucidating the complex interactions among keywords. A.csv Excel file has been submitted to the Biblioshiny platform. Comma-separated values files (.csv) and Portable Network Graphics files (.png) were obtained and used for data analysis following the study’s objectives. We used VOSviewer (version 1.6.20) (www.vosviewer.com) to illustrate extensive data on tasar silkworm (24, 25). The VOSViewer program, designed for the generation and interpretation of bibliometric maps, was used to assess the worldwide publishing landscape. It allows users to construct and display a network or relationship via a text-mining technique when referencing an article. It may delineate comprehensive articles and publications using many display choices and features, like zooming, scrolling, and searching (26). **Supplementary Table 1** displays the fundamental bibliometric information on tasar silkworm, obtained by the Biblioshiny program.

## 3 Analysis of the data

### 3.1 Annual publication growth

**Figure 1:**
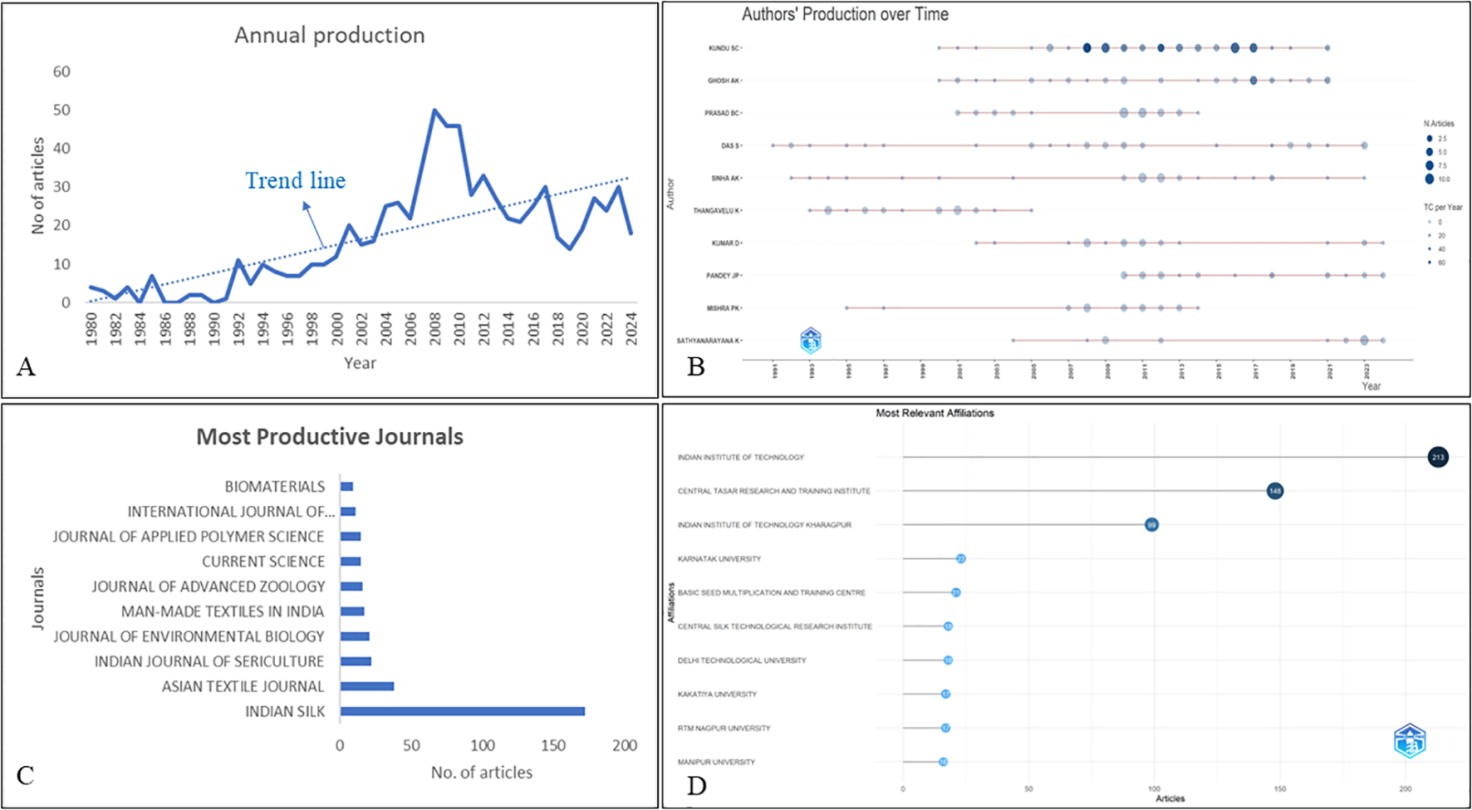
**(A)** The annual publication growth trend of Tasar silkworm-based research. **(B)** The most relevant authors and their production over time. **(C)** Top ten most productive journals. **(D)** Top ten relevant affiliations contributed to Tasar silkworm research.

**Figure 1A** illustrates the temporal increase of papers in a bibliometric investigation of the Tasar silkworm. The peak observed between 2007 and 2010, with the highest count in 2008 (50 articles), aligns with a period of increased global interest in sericulture due to advancements in molecular biology, genetics, and biotechnology. During this phase, research was focused on improving Tasar silkworm breeding, disease resistance, and productivity, which led to increased publications. Additionally, governmental and institutional funding in developing sustainable silk production practices, particularly in India and China, further contributed to this surge. However, the decline in publications post-2010, particularly after 2018, may be due to several scientific and industrial shifts. Firstly, research in sericulture may have reached a saturation point in fundamental aspects, with fewer groundbreaking discoveries leading to a decline in new studies. Secondly, shifting research priorities towards more modern biotechnological and genomic interventions in commercial silkworm species such as *Bombyx mori* may have diverted focus away from Tasar silkworms. Additionally, climate change and deforestation have impacted the availability of host plants like *Terminalia arjuna* and *T. tomentosa*, affecting Tasar silkworm rearing and reducing interest in this field. Furthermore, declining governmental investments in traditional sericulture research, as newer economic sectors such as synthetic fiber industries gain prominence, might have played a role in the reduced number of studies in recent years. Hence, while Tasar silkworm research peaked due to scientific and industrial relevance, its decline is largely due to evolving research interests, funding shifts, and ecological challenges.

### 3.2 The most relevant authors

The dataset (**Supplementary Table 2**) provides insights into the research productivity and impact of authors in Tasar silkworm studies. Kundu S.C. leads with 71 publications, 28,274 citations (26,837 excluding self-citations), and the highest h-index of 76 (71 excluding self-citations), with productivity peaks in 2008, 2012, and 2016. Ghosh A.K. follows with 38 publications, 2,616 citations (2,450 excluding self-citations), and an h-index of 30, peaking in 2017. Prasad B.C. (36 publications, h-index 8) was highly active in 2010, while Das S. (24 publications, h-index 8) peaked in 2010 and 2018. Pandey J.P. (26 publications, h-index 14) and Sinha A.K. (29 publications, h-index 6) peaked in 2018 with 2 papers and 74 citations each. Other contributors, including Kumar D. (20 publications, h-index 15), Mishra P.K. (24 publications, h-index 6), and Sathyanarayana K. (24 publications, h-index 4), maintained steady outputs. Fluctuations in research activity suggest citation impact was shaped by advancements in sericulture, funding trends, and evolving research priorities, highlighting quality over quantity in long-term scholarly influence. **Figure 1B** illustrates the leading authors and their output over time.

### 3.3 The most productive journals

**Figure 1C** highlights the 10 highest-producing journals on Tasar silkworm research, showing a steady increase in output. *Indian Silk* exhibited the most growth, rising from zero articles in 1980 to 172 annually since 2017. *Asian Textile Journal* expanded from one article in 1992 to 38 in 2024, while *Indian Journal of Sericulture*, *Journal of Environmental Biology*, and *Man-Made Textiles in India* reached 22, 21, and 17 articles per year, respectively. However, impact metrics (**Supplementary Table 3**) reveal that higher production does not always equate to greater scholarly influence. Among analyzed journals, *Biomaterials* (IF: 12.8, SJR: 3.016, h-index: 435) exhibits the strongest citation impact, followed by the *International Journal of Biological Macromolecules* (IF: 7.7, SJR: 1.25, h-index: 191) and the *Journal of Applied Polymer Science* (IF: 2.7, SJR: 0.56, h-index: 193). In contrast, high-output journals like *Indian Silk*, *Asian Textile Journal*, and *Indian Journal of Sericulture* lack impact factors and have lower h-indices (6, 11, and 10, respectively). The *Journal of Environmental Biology* (IF: 0.6, h-index: 61) and *Current Science* (IF: 1.1, h-index: 137) hold moderate influence. These findings suggest that while research production has increased significantly, scholarly impact depends more on journal reputation, research scope, and citation influence rather than sheer volume. High-impact research is often concentrated in specialized, well-ranked journals rather than the most productive ones, reflecting the growing interest in textiles, biomaterials, and environmental biology.

### 3.4 The most relevant institutions

This study examined the published output of organizations or authors’ affiliations involved in Tasar silkworm research, as seen in **Figure 1D**. Among all the institutions, the Indian Institute of Technology (IIT) ranked first, with 213 publications, showcasing its significant contribution to textile and sericulture research. Individually, the Central Tasar Research and Training Institute (CTRTI) secured the second position with 148 publications, emphasizing its dedicated focus on Tasar silkworm breeding, disease management, and sustainable silk production. Other notable contributors include IIT Kharagpur (99 publications), Karnatak University (23), and the Basic Seed Multiplication and Training Centre (21), a key unit under the Basic Tasar Silkworm Seed Organisation (BTSSO). Additionally, regional institutions such as Manipur University (16) and RTM Nagpur University (17) have played a vital role in advancing sericulture research, supporting localized scientific and industrial efforts.

### 3.5 Country scientific production

**Figure 2:**
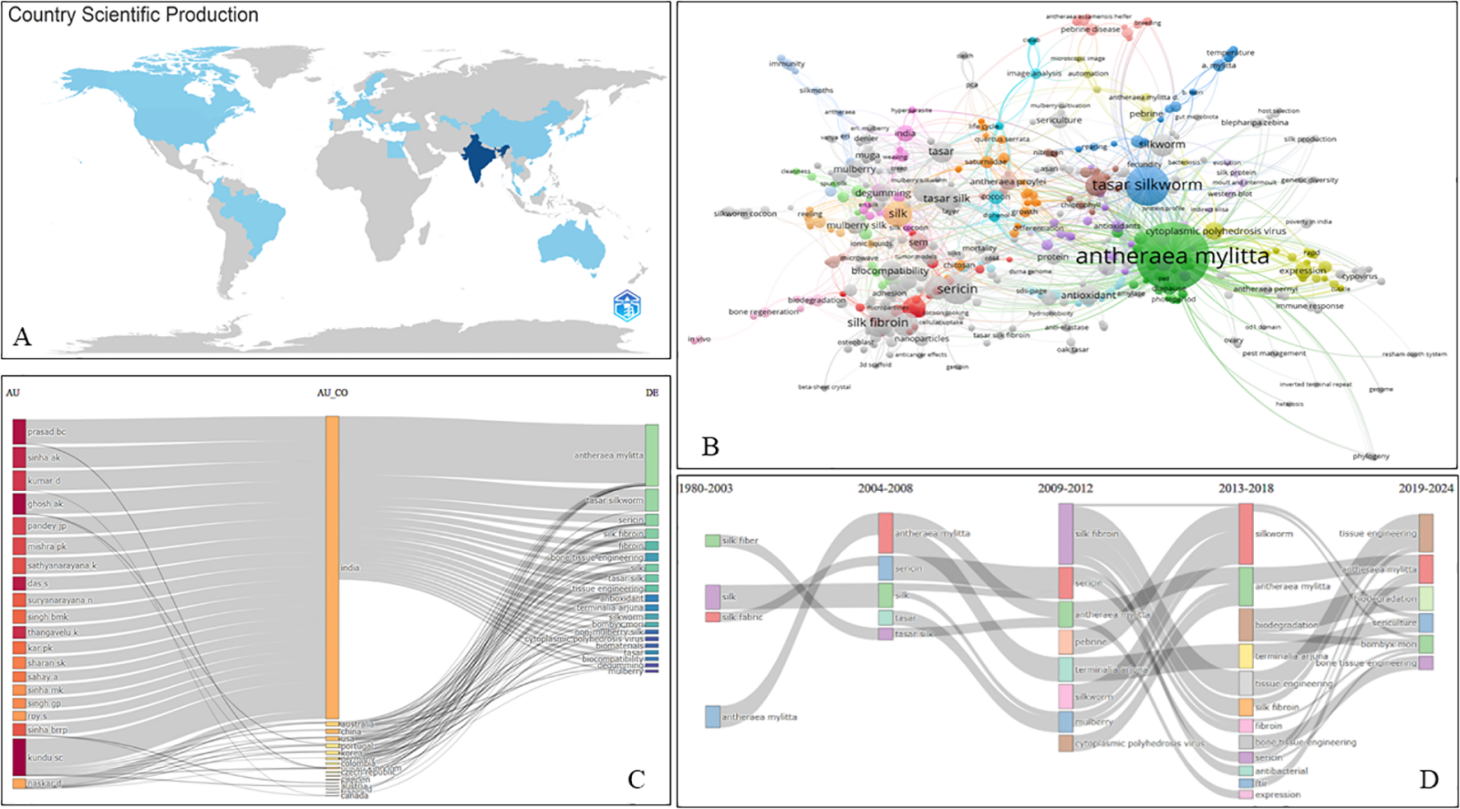
**(A)** Country scientific production. **(B)** Co-occurrence of keywords in Tasar silkworm related research. **(C)** Relationship found in Tasar silkworm-based research between authors, countries, and keywords. **(D)** Thematic evolution in Tasar silkworm-based research.

**Figure 2A** highlights global contributions to Tasar silkworm research, with India leading significantly, producing 331 articles (44.7% of total publications). India’s research output surged from 6 papers in 1980 to 2,404 in 2024, driven by governmental and institutional support. China and the USA follow with 9 articles each, while the UK contributes 7, indicating growing international interest. India’s dominance is evident in its Single Country Publications (SCP) of 304, compared to just 27 Multiple Country Publications (MCP) (8.2%), suggesting domestic institutions like the Central Tasar Research and Training Institute and IITs primarily drive research. In contrast, Portugal, Egypt, and Korea (100% MCP) and countries like Sweden (75%), the UK (71.4%), and the USA (44.4%) rely more on international collaborations. India’s research expansion aligns with its focus on genetic improvements, disease resistance, and silk quality enhancement, vital for rural livelihoods and economic growth. Institutions such as Basic Seed Multiplication and Training Centres further strengthen advancements in breeding techniques, cocoon productivity, and sericulture sustainability. While India remains the global hub, greater international collaborations could enhance innovation, integrating advanced biotechnological approaches for improved silk production. Further details on country collaboration and research trends are provided in **Supplementary Figure 2**.

### 3.6 The most frequent words and relationship with authors and countries

**Figure 2B** highlights key research themes in Tasar silkworm studies, emphasizing species, processes, and applications. *Antheraea mylitta*, a dominant species in non-mulberry silk production, appears 123 times in keyword analysis. Research focuses on fibroin (20 occurrences) and sericin (24 occurrences), valued for their roles in biomaterials and tissue engineering (15). The growing interest in non-mulberry silk (10) stems from its biodegradability, superior mechanical properties, and biomedical applications, including bone tissue engineering (12), wound healing (3), and regenerative medicine (3). Additionally, Tasar silk’s biocompatibility (10), scaffold potential (6), and osteogenesis support (4) highlight its significance. Studies also examine sericulture health, focusing on diseases like Cytoplasmic Polyhedrosis Virus (8) and Pebrine (6). The presence of antioxidants (10) and antibacterial activity (6) enhances its use in drug delivery and antimicrobial wound dressings. Advances in genomic sequencing, proteomics, and imaging techniques are shaping the field, shifting from traditional sericulture to modern interdisciplinary research.

**Figure 2C** presents Sankey diagrams illustrating global research contributions. The three-field plot connects nations, authors, and keywords, where India and China emerge as leading contributors. The diagram visually represents the top 20 productive countries and authors, with link thickness indicating the strength of collaborations and research focus. This analysis provides insights into emerging trends and key research networks in Tasar silkworm studies.

### 3.7 Thematic evolution

The thematic evaluation of *Antheraea mylitta* research from 1980 to 2024 reveals a significant shift from fundamental silk characterization to advanced biomedical applications (**Figure 2D**). Between 1980 and 2003, studies primarily focused on Tasar silkworm (*Antheraea mylitta*), silk properties, and fibroin composition, as reflected in the strong linkages between keywords such as “Tasar silk” and “fibroin.” From 2004 to 2012, research expanded to include sericin and its potential applications, with growing connections to *Terminalia arjuna*, indicating interest in natural antioxidants and eco-friendly processing techniques. The period between 2013 and 2018 saw a shift toward biomedical applications, with increasing references to tissue engineering, antibacterial properties, and biocompatibility. Notably, keywords like “scaffolds,” “osteogenesis,” and “hydrogel” emerged, suggesting a growing interest in biomaterials for regenerative medicine. From 2019 to 2024, the research further advanced into biodegradation, oxidative stress, and hemolymph studies, with *Antheraea mylitta* being explored for its antioxidant potential. The inclusion of “bone tissue engineering” and “biodegradation” in recent years highlights the transition of Tasar silk from a textile fiber to a high-value biomaterial with applications in medicine and environmental sustainability. This timeline underscores the evolving role of *Antheraea mylitta* research, from traditional sericulture to cutting-edge biomaterial science.

## 4 Conclusion and future directions

The bibliometric analysis of Tasar silkworm (*Antheraea mylitta*) research (1980–2024) reveals key trends and evolving scientific priorities. Early studies (1980–2003) focused on silk properties and fibroin composition, while 2004–2012 expanded to sericin’s eco-friendly and antioxidant applications. Between 2013–2018, research shifted to biomedical applications, emphasizing scaffolds, osteogenesis, and tissue engineering. The latest phase (2019–2024) explores biodegradation, oxidative stress, and hemolymph analysis, positioning Tasar silk as a biomaterial for medical and environmental applications. Research productivity peaked (2007–2010) alongside advancements in molecular biology and genetics, but a decline post-2018 suggests a shift toward commercially dominant species like *Bombyx mori*. Environmental factors, including deforestation and climate change, may also influence research trends. India leads Tasar silkworm studies, contributing 44.7% of global publications, driven by IITs, CTRTI, and universities, with limited international collaboration. In contrast, China, the USA, and the UK engage primarily in collaborative research. While high-output journals like *Indian Silk* and *Asian Textile Journal* contribute to research volume, high-impact journals (*Biomaterials*, *Journal of Applied Polymer Science*) focus on biomaterials and biotechnology, reflecting a shift from sericulture to interdisciplinary applications. Keyword and thematic analysis confirm Tasar silk’s transition from textile research to biomedical and sustainable technology applications. Increasing references to antioxidants, antibacterial properties, and tissue engineering highlight its potential in biomedicine, drug delivery, and regenerative medicine.

Building upon the insights gained from this bibliometric analysis, several key areas emerge as promising directions for future research and development in Tasar silkworm studies.

1. Genomic and Biotechnological Advancements: Future studies should employ genomic and proteomic profiling of *Antheraea mylitta* to enhance disease resistance, genetic traits, and silk quality. Advanced tools like CRISPR gene editing and transcriptome analysis can facilitate precision breeding and molecular interventions.
2. Biomedical Applications: Expanding research on Tasar silk fibroin and sericin for wound healing, drug delivery, and tissue engineering is crucial. Integrating nanotechnology and hydrogels may unlock novel biomedical and regenerative medicine applications.
3. Sustainable Processing: Research should focus on eco-friendly silk processing, including biodegradable coatings, non-toxic dyeing, and sustainable sericulture practices, enhancing commercial viability while reducing environmental impact.
4. Global Collaboration & Interdisciplinary Research: Strengthening international collaborations can provide access to advanced technologies and funding. Partnerships among sericulture scientists, material engineers, and biomedical researchers can drive innovation in Tasar silk applications.
5. Climate Resilience Strategies: Addressing climate change effects on Tasar silkworms and host plants (*Terminalia arjuna* & *T. tomentosa*) is essential. Developing climate-resilient breeding programs and adaptive rearing methods will ensure sustainable silk production.
6. Industrial & Commercial Applications: Strengthening industry-academia partnerships can accelerate commercialization. Exploring Tasar silk-based composites, functional textiles, and bio-inspired materials could expand industrial and market-driven innovations.

By integrating these future research directions, Tasar silkworm studies can continue to evolve beyond traditional sericulture, positioning *Antheraea mylitta* as a key player in biomaterial science, sustainable textiles, and biomedical engineering. The convergence of advanced biotechnology, interdisciplinary collaborations, and sustainable practices will define the next phase of Tasar silk research, ensuring its continued relevance in both scientific and industrial domains.

## Data Availability

The datasets presented in this study can be found in online repositories. The names of the repository/repositories and accession number(s) can be found in the article/**Supplementary Material**.
