# Supplementary material for: Bibliometric trends and patterns in Tasar silkworm (Antheraea mylitta) research: a data report (1980–2024)
Source: Front Insect Sci. 2025 Apr 30;5:1533267. doi: 10.3389/finsc.2025.1533267 (PMC12075178; doi:10.3389/finsc.2025.1533267)
Supplement: Supplementary file 1 [file Table1.docx]

| **Description** | **Results** |
| --- | --- |
| **Main information about data** | |
| Timespan | 1980:2024 |
| Sources (Journals, Books, etc) | 263 |
| Documents | 741 |
| Annual Growth Rate % | 3.48 |
| Document Average Age | 15.5 |
| Average citations per doc | 13.76 |
| References | 16379 |
| **Document contents** | |
| Keywords Plus (ID) | 4186 |
| Author's Keywords (DE) | 1363 |
| **Authors** | |
| Authors | 1267 |
| Authors of single-authored docs | 54 |
| **Authors collaboration** | |
| Single-authored docs | 69 |
| Co-Authors per Doc | 3.78 |
| International co-authorships % | 7.692 |
| **Document types** | |
| Article | 686 |
| Book | 1 |
| Book chapter | 9 |
| Conference paper | 14 |
| Conference review | 1 |
| Letter | 1 |
| Note | 5 |
| Review | 20 |
| Short survey | 4 |

**Supplementary Table 1.** Main Information
