# Supplementary material for: Bibliometric trends and patterns in Tasar silkworm (Antheraea mylitta) research: a data report (1980–2024)
Source: Front Insect Sci. 2025 Apr 30;5:1533267. doi: 10.3389/finsc.2025.1533267 (PMC12075178; doi:10.3389/finsc.2025.1533267)
Supplement: Supplementary file 2 [file Table2.docx]

| **Researcher Name** | **Total Citations** | **Citations Excl. Self** | **H-Index** | **H-Index Excl. Self** | **No. of Documents** |
| --- | --- | --- | --- | --- | --- |
| Kundu S.C | 28274 | 26837 | 76 | 71 | 71 |
| Ghosh A. K | 2616 | 2450 | 30 | 30 | 38 |
| Prasad B.C | 196 | 159 | 8 | 7 | 36 |
| Das S | 344 | 307 | 8 | 8 | 24 |
| Sinha A. K | 157 | 143 | 6 | 6 | 29 |
| Thangavelu K | 344 | 336 | 8 | 7 | 29 |
| Kumar D | 680 | 647 | 15 | 15 | 20 |
| Pandey J. P | 481 | 415 | 14 | 13 | 26 |
| Mishra P. K | 104 | 71 | 6 | 5 | 24 |
| Sathyanarayana K | 74 | 49 | 4 | 3 | 24 |

**Supplementary table 2**
