# Supplementary material for: Bibliometric trends and patterns in Tasar silkworm (Antheraea mylitta) research: a data report (1980–2024)
Source: Front Insect Sci. 2025 Apr 30;5:1533267. doi: 10.3389/finsc.2025.1533267 (PMC12075178; doi:10.3389/finsc.2025.1533267)
Supplement: Supplementary file 3 [file Table3.docx]

| **Journal Name** | **Impact Factor (IF)** | **H Index** | **Quartile** | **Scimago Journal Rank (SJR)** |
| --- | --- | --- | --- | --- |
| Indian Silk | N/A | 6 | N/A | 0.104 |
| Asian Textile Journal | N/A | 11 | Q4 | 0.1 |
| Indian Journal of Sericulture | N/A | 10 | N/A | 0.11 |
| Journal of Environmental Biology | 0.6 | 61 | Q3 | 0.235 |
| Man-Made Textiles in India | N/A | 11 | Q4 | 0.126 |
| Journal of Advanced Zoology | N/A | 11 | Q4 | 0.1 |
| Current Science | 1.1 | 137 | Q2 | 0.24 |
| Journal of Applied Polymer Science | 2.7 | 193 | Q2 | 0.56 |
| International Journal of Biological Macromolecules | 7.7 | 191 | Q1 | 1.25 |
| Biomaterials | 12.8 | 435 | Q1 | 3.016 |

**Supplementary table 3**
