# Supplementary figures and images for: Bibliometric trends and patterns in Tasar silkworm (Antheraea mylitta) research: a data report (1980–2024)
Source: Front Insect Sci. 2025 Apr 30;5:1533267. doi: 10.3389/finsc.2025.1533267 (PMC12075178; doi:10.3389/finsc.2025.1533267)

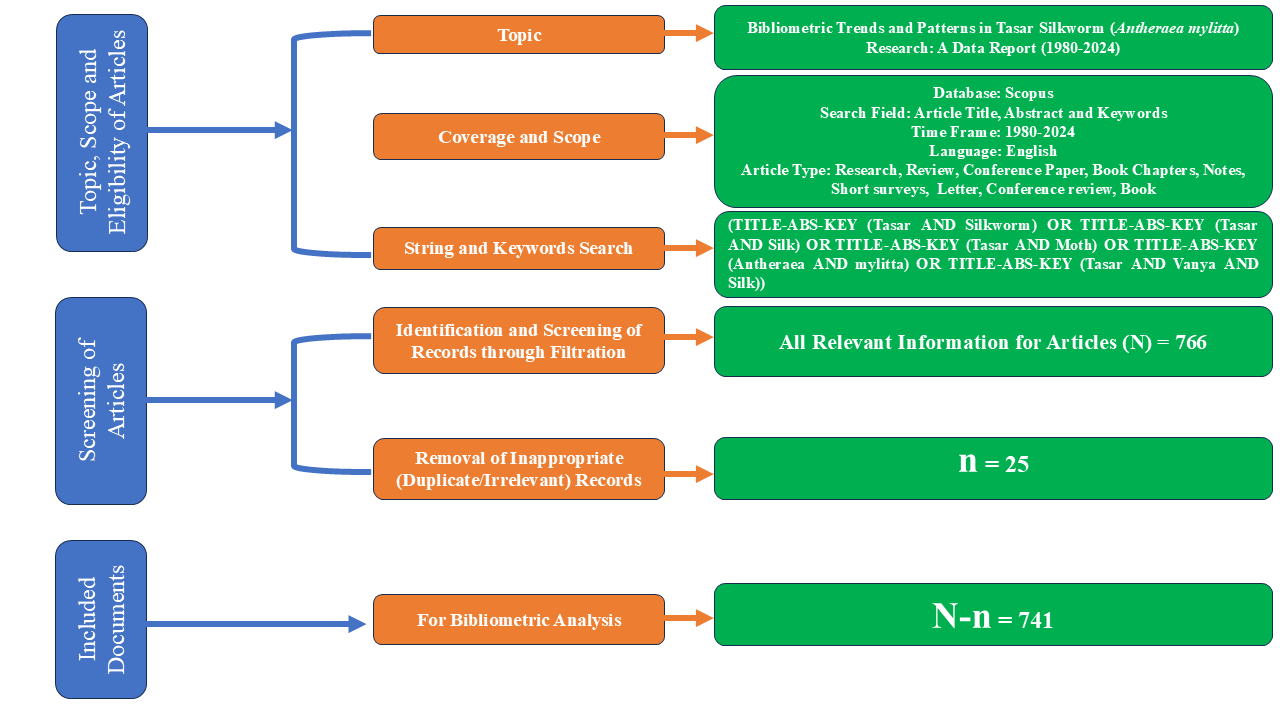

Supplement: Supplementary file 4 [file Image1.tif]

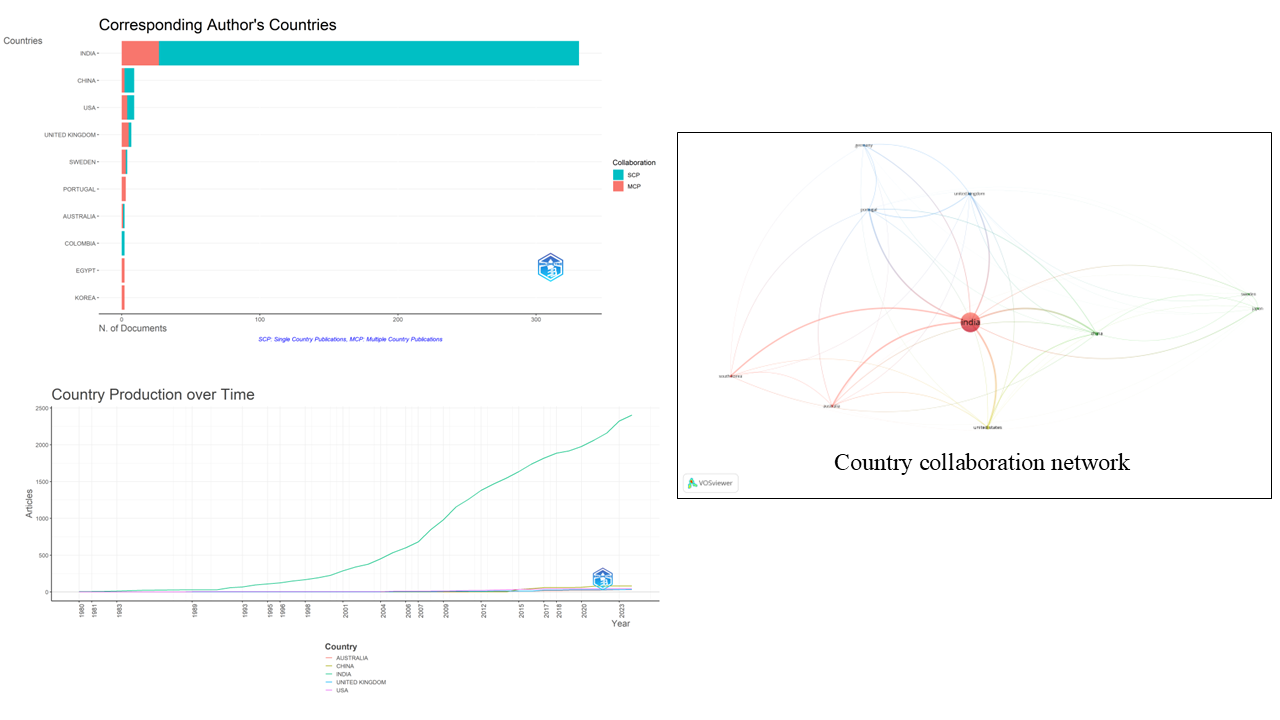

Supplement: Supplementary file 5 [file Image2.tif]
